# A Novel, Multi-Faceted Perception of Lactate in Neurology

**DOI:** 10.3389/fnins.2020.00460

**Published:** 2020-05-15

**Authors:** Shayne Mason

**Affiliations:** Human Metabolomics, North-West University, Potchefstroom, South Africa

**Keywords:** lactate, neurology, traumatic brain injury (TBI), neurodegenerative disease, tuberculous meningitis (TBM)

## Introduction

Lactate has become one of the most re-evaluated metabolites in energy metabolism, particularly as a shuttle in neuroenergetics (Mason, 2017; Ferguson et al., 2018). In a comprehensive review in 2018, Brooks, one of the originators of the “lactate shuttle” concept, discusses the roles that lactate plays in the delivery of oxidative, and gluconeogenic substrates, as well as in cell signaling (Brooks, 2018). Brooks also appraises clinical studies that feature lactate to treat pro-inflammatory conditions, as well as reports on historic and recent studies of lactate metabolism and shuttling. A pioneering astrocyte–neuron lactate shuttle (ANLS) hypothesis was first proposed in 1994 by Pellerin and Magistretti for homeostatic conditions within the brain (Pellerin and Magistretti, 1994). The ANLS hypothesis has withstood challenges for 25 years but remains controversial as scientists continue to debate its validity (Bak and Walls, 2018; Barros and Weber, 2018). I believe, however, that the ANLS hypothesis can be extended beyond homeostatic conditions into neuropathophysiological states. The focus of this short opinion paper is to highlight studies over the past 2 years that support the notion that the route of lactate, as it acts as a shuttle in the brain, in neuropathophysiological states is emerging as crucial in neuroenergetics. This behavior is reviewed in more detail by Mason (2017). Further, this paper presents recent experimental evidence that has emerged supporting an extension of the ANLS—the newly postulated astrocyte–microglia lactate shuttle (AMLS) hypothesis (Mason et al., 2015), as described for the infectious, chronic neuroinflammatory disease tuberculous meningitis (TBM).

## Multifaceted Role of Lactate in Neurometabolism

The neuroprotective role of lactate, particularly during cerebral ischemia (Castillo et al., 2015), is well-substantiated in the literature (Mason, 2017). Recent studies on central nervous system injury show that induction of glycolytic flux in astrocytes generates an accumulation of lactate in these cells. It is the release of lactate into the extracellular space that incites lactate-mediated neuroprotection (Huang et al., 2019; Tauffenberger et al., 2019; Vohra et al., 2019; Vohra and Kolko, 2020).

New insights into lactate cellular compartmentation have been gained by measuring great variations in lactate diffusion properties *in vivo* (Ligneul et al., 2019), which show that intracellular lactate is predominantly present within astrocytes under control conditions, but comes to be predominate in neurons during “pure” astrocyte reactivity (in the absence of both neuronal death and microglial activation). The novel results of Ligneul et al. indicate extensive remodeling of lactate metabolism, as lactate compartmentation is tightly linked to the ANLS mechanism.

When considering neuroenergetics and the importance of lactate supply to sustain neuronal activity, it has been shown (Mazuel et al., 2017) that knockdown of the protein monocarboxylate transporter 2 (MCT2) in the rat somatosensory cortex prevents both the transient lactate rise caused by whisker stimulation as well as the concomitant blood oxygen level-dependent response, suggesting an altered neuronal response if lactate supply to neurons is impaired. It has also been shown that enhancing the expression of MCT2—using the recombinant *Lonomia obliqua* Stuart-factor activator—together with providing lactate is beneficial for cultured neurons against serum deprivation stress (Alvarez-Flores et al., 2019). Alvarez-Flores et al. proposed that this might represent a novel therapeutic approach based on the possibility of boosting brain energy metabolism.

Computational models of neurostimulation show rapid shuttling of lactate from astrocytes into the extracellular space for use by neurons (Coggan et al., 2018). Glycogen in astrocytes is needed for rapid lactate production by astrocyte glycolysis; however, this can be bypassed by nitric oxide (NO) production, which results in immediate and reversible intracellular glucose depletion and modulation of the extracellular lactate reservoir originating from astrocytes (San Martín et al., 2017). The causal relationship between the NO of the cerebrospinal fluid (CSF) and lactate of the CSF is a phenomenon that, in my opinion, needs investigation in neuropathophysiological states.

## Emerging Support for the New AMLS Hypothesis in TBM

The new AMLS hypothesis (a derivative of the ANLS postulate) is based upon an untargeted ^1^H-NMR metabolomics study on CSF from a pediatric cohort of TBM patients (Mason et al., 2015). The AMLS model requires that the increased levels of lactate that are produced within the astrocytes are transported into the extracellular space. Instead of being shuttled to the neurons as described in the ANLS, the lactate is then redirected toward the mitochondria of microglia for increased production of mitochondrial energy. Increased mitochondrial activity within the microglia leads to greater electron leakage and subsequently raised production of free radicals. One such free radical—superoxide—reacts with NO to produce reactive nitrogen species (RNS) (Valerio and Nisoli, 2015). Reactive oxygen species (ROS) and RNS are an essential defense produced by microglia to deal with the immediate injury/infection during TBM; however, chronic neuroinflammation leads to gliosis due to persistent ROS/RNS. Recently, using the same analytical platform and similar sample size, but based upon a cohort of adults, Zhang et al. (2019) found metabolic markers distinguishing between TBM and controls consistent with our original data. This adult cohort demonstrated CSF lactate to be on average 4.2 times higher in TBM cases than in controls. In our pediatric cohort (Mason et al., 2015), we similarly found the CSF lactate levels to be elevated—an average of 3.2 times higher in TBM cases than in controls.

A prospective hospital-based cross-sectional study (Siddiqi et al., 2018) of adults over 15 years identified 55 patients, out of 220 meningo-encephalitis cases, with TBM of statistically significant class differences, with possible, probable, and definite classes (based upon a uniform definition given by Marais et al., 2010) having mean CSF lactate levels of 3.7 ± 1.6, 6.1 ± 2.8, and 8.1 ± 1.7 mM, respectively. Thus, mean CSF lactate levels increased corresponding to improvement in the certainty of TBM diagnosis, indicating that CSF lactate is therefore apparently linked to the severity of TBM. Mean CSF lactate values were also increased (to 1.2, 2.4, and 4.4 mM) in severity of clinical TBM stages 1, 2, and 3, respectively. Hence, CSF lactate increases with severity of infections that cause chronic neuroinflammation, significantly more so than in cases of acute viral meningitis (Li et al., 2017; Zhang et al., 2019). Siddiqi et al. correctly state that there is a “*scarcity of studies of CSF lactate in TBM and its role as a diagnostic and prognostic marker has not been elucidated*.” Therefore, further research should be conducted to validate CSF lactate as a diagnostic and prognostic marker in TBM.

## Diverse Neurology, But a Common Factor—Perturbed Lactate

Biochemical disturbances, and their causal effects, within the brain can be temporal, diverse, and severe. Using Cx43 cKO^GFAP^ mice, Clasadonte et al. (2017) demonstrated that delivery of lactate from astrocytes to neurons is required for normal orexinergic neuronal activity. Insufficient supply of lactate from astrocytes leads to a marked and selective loss of orexin neurons in the lateral hypothalamic area. This has been identified as one of the main causes of narcolepsy (Peyron et al., 2000; Thannickal et al., 2000).

In an experiment using neonatal piglets to simulate newborn hypoxic-ischemic encephalopathy (Zheng and Wang, 2018), uptake values of lactate (peaking at 2–6 h) and glucose (peaking at 6–12 h) in the basal ganglia and basal ganglia/occipital cortex increased following hypoxic-ischemic reperfusion brain injury. Lactate levels remained higher than controls for up to 72 h and, histologically, astrocyte damage occurred earlier and more severely than neuronal damage. In another study, induced tonic–clonic seizure in rats (Oses et al., 2019) was associated with increased glucose and lactate levels immediately within the brain, with the CSF lactate/glucose ratio remaining >2:1 for 24 h except at the 10 min mark, where it was 1:1. However, mitochondria were not able to increase energy production due to an uncoupling between mitochondrial oxygen consumption and ATP synthesis via FoF1-ATP synthase, thereby affecting cell viability. Thus, across diverse types of neurology, recent studies continue to demonstrate that lactate remains a focal point of biological importance.

## Lactate is Indispensable in Neurodegenerative Diseases

Neurodegenerative diseases, which is more complicated the more deeply we explore them, rely upon a common simple metabolite—lactate. Research on Alzheimer's disease (AD), using the APP/PS1 mouse model and ^13^C-NMR metabolomics, has revealed a decrease in the lactate–alanine shuttle in the brain (Zhou et al., 2018), limiting the source of ammonia nitrogen from alanine that is used in the glutamate–glutamine cycle (Schousboe et al., 2003). Immunohistochemistry showed that APP/PS1 mice exhibited less MCT2 staining in the cerebral cortex and hippocampus than the WT mice (Lu et al., 2019). Reduced lactate and downregulated MCT2 in the cerebral cortex and hippocampus can decrease the lactate content in the neurons, associated with an energy crisis, downregulated expression of long-term memory-related proteins, and consequently cause memory decline in AD. Interestingly, curcumin treatment of APP/PS1 mice, which presented significantly increased lactate content and markedly higher MCT2 protein levels in the cerebral cortex and hippocampus, correlated with improvement of memory (Lu et al., 2019). Furthermore, the observations of Harris et al. (2019) suggest that lactate production may be required for memory acquisition but not retrieval. Hence, boosting the neuroprotective properties of astrocytes (by boosting lactate production of astrocytes) has potential applications in delaying the onset and progression of AD, as originally proposed by Demetrius et al. (2015) and summarized in a review by Zulfiqar et al. (2019). Furthermore, glucose hypometabolism is typically observed in AD. Based upon recent ^18^FDG-PET imaging studies (Zimmer et al., 2017; Carter et al., 2019), a deficit in glucose metabolism in astrocytes (but not in neurons) has been observed that would reduce lactate supply to neurons and render them more vulnerable.

Another important lactate shuttling system exists between oligodendrocytes and axons, first demonstrated by Lee et al. (2012). It has also been shown that oligodendrocytes prefer lactate over glucose as a substrate for myelin production (Rinholm et al., 2011). The disruption of this oligodendrocyte–axon lactate shuttle contributes to the pathogenesis of another form of neurodegenerative disease—multiple sclerosis (MS). In a 2018 review Rosko et al. describe how lactate is critical for oligodendrocyte function and oligodendrocyte–axon coupling (Rosko et al., 2018). Evidence that lactate is an important metabolite during the course of AD and MS pathogenesis is becoming increasingly better understood. The potential therapeutic role of lactate in neurodegenerative diseases is one that awaits more attention.

## Therapeutic Role of Lactate in Acute Brain Injuries

The therapeutic role of lactate in acute brain injuries such as subarachnoid hemorrhage and traumatic brain injury (TBI)—identified by a high lactate/pyruvate ratio despite adequate brain glucose and oxygenation—was recently reviewed by Killen et al. (2019). Two TBI studies in 2018 revealed: (1) constant perfusion of 8 mM sodium 3-^13^C lactate over 24 h using microdialysis catheters in the brains of nine patients with severe TBI exhibited ^13^C-glutamine enrichment above the non-TBI control range, suggesting lactate oxidative metabolism as a TBI “emergency option” (Jalloh et al., 2018). (2) Transcriptional analysis of gene expression modulation in neurons evoked by exposure to L-lactate indicated that lactate effectively regulates activity-dependent and synaptic genes and highlighted new signaling effects of lactate in plasticity and neuroprotection (Margineanu et al., 2018). A study by Wolahan et al. (2018), in which moderate, constant intravenous infusions of 2 mM sodium L-lactate for TBI patients (over a median time of 137 h post-injury) showed arterial lactate concentrations increased from 0.92 to 1.84 mM, no changes in systemic glucose, decreased intracranial pressure of 3.6 mmHg after 1 h and increased cerebral uptake of lactate and raised concentrations of systemic metabolites. Wolahan et al. concluded that at moderate infusion rates and variable changes to the patients' systemic lactate that the net balance of cerebral lactate uptake and release shifts toward uptake, which could improve cerebral neuroenergetics by generating additional ATP to fuel the cellular recovery processes. Further studies, such as those by Ligneul et al. (2019), are needed to determine compartmentalization of cerebral lactate during TBI.

These new studies contribute to the concept that lactate can be used as a putative treatment for TBI patients (Quintard et al., 2016; Mason, 2017). However, a limitation when involving human TBI studies, as expressed by Jalloh et al. (2018), is that there are no truly normal controls and that during treatment, surgery itself may constitute some degree of trauma (altered metabolic profile) that cannot be ruled out.

## Final Thought

Lactate is a chemically simple metabolite, yet its dynamics within shuttling systems in neurology is profoundly complex. It has been shown (Mason et al., 2016) that highly lactic acidotic CSF from infants and children with confirmed TBM exhibits only the L-enantiomer—meaning that it is a response solely by the host to the infection. In TBM cases, it appears that lactate is a crucial energy substrate, used preferentially over glucose by microglia, and exhibits neuroprotective capabilities. Lactate levels should be carefully considered by clinicians during diagnosis, especially when considering communicable neuroinflammatory diseases.

## Author Contributions

The author confirms being the sole contributor of this work and has approved it for publication.

## Conflict of Interest

The author declares that the research was conducted in the absence of any commercial or financial relationships that could be construed as a potential conflict of interest.

## References

[B1] Alvarez-Flores M. P. Hébert A. Gouelle C. Geller S. Chudzinski-Tavassi A. M. Pellerin L. (2019). Neuroprotective effect of rL osac on supplement-deprived mouse cultured cortical neurons involves maintenance of monocarboxylate transporter MCT 2 protein levels. J. Neurochem. 148, 80–86. 10.1111/jnc.14617 30347438

[B2] Bak L. K. Walls A. B. (2018). CrossTalk opposing view: lack of evidence supporting an astrocyte-to-neuron lactate shuttle coupling neuronal activity to glucose utilisation in the brain. J. Physiol. 596, 351–353. 10.1113/JP274945 29292507 PMC5792606

[B3] Barros L. F. Weber B. (2018). CrossTalk proposal: an important astrocyte-to-neuron lactate shuttle couples' neuronal activity to glucose utilisation in the brain. J. Physiol. 596, 347–350. 10.1113/JP274944 29292516 PMC5792514

[B4] Brooks G. A. (2018). The science and translation of lactate shuttle theory. Cell Metab. 27, 757–785. 10.1016/j.cmet.2018.03.008 29617642

[B5] Carter S. F. Herholz K. Rosa-Neto P. Pellerin L. Nordberg A. Zimmer E. R. (2019). Astrocyte biomarkers in Alzheimer's disease. Trends Mol. Med. 25, 77–95. 10.1016/j.molmed.2018.11.006 30611668

[B6] Castillo X. Rosafio K. Wyss M. T. Drandarov K. Buck A. Pellerin L.. (2015). A probable dual mode of action for both L-and D-lactate neuroprotection in cerebral ischemia. J. Cerebr. Blood F. Met. 35, 1561–1569. 10.1038/jcbfm.2015.115 26036941 PMC4640320

[B7] Clasadonte J. Scemes E. Wang Z. Boison D. Haydon P. G. (2017). Connexin 43-mediated astroglial metabolic networks contribute to the regulation of the sleep-wake cycle. Neuron 95, 1365–1380. 10.1016/j.neuron.2017.08.022 28867552 PMC5617118

[B8] Coggan J. S. Keller D. Calì C. Lehväslaiho H. Markram H. Schürmann F.. (2018). Norepinephrine stimulates glycogenolysis in astrocytes to fuel neurons with lactate. PLoS Comput. Biol. 14:e1006392. 10.1371/journal.pcbi.1006392 30161133 PMC6160207

[B9] Demetrius L. A. Magistretti P. J. Pellerin L. (2015). Alzheimer's disease: the amyloid hypothesis and the Inverse Warburg effect. Front. Physiol. 5:522. 10.3389/fphys.2014.00522 25642192 PMC4294122

[B10] Ferguson B. S. Rogatzki M. J. Goodwin M. L. Kane D. A. Rightmire Z. Gladden L. B. (2018). Lactate metabolism: historical context, prior misinterpretations, and current understanding. Eur. J. Appl. Physiol. 118, 691–728. 10.1007/s00421-017-3795-6 29322250

[B11] Harris R. A. Lone A. Lim H. Martinez F. Frame A. K. Scholl T. J. (2019). Aerobic glycolysis is required for spatial memory acquisition but not memory retrieval in mice. eNeuro 6, 389–418. 10.1523/ENEURO.0389-18.2019 PMC6390195 30809587

[B12] Huang L. Nakamura Y. Lo E. H. Hayakawa K. (2019). Astrocyte signaling in the neurovascular unit after central nervous system injury. Int. J. Mol. Sci. 20:282. 10.3390/ijms20020282 30642007 PMC6358919

[B13] Jalloh I. Helmy A. Howe D. J. Shannon R. J. Grice P. Mason A.. (2018). A comparison of oxidative lactate metabolism in traumatically injured brain and control brain. J. Neurotrauma 35, 2025–2035. 10.1089/neu.2017.5459 29690859 PMC6098406

[B14] Killen M. J. Giorgi-Coll S. Helmy A. Hutchinson P. J. Carpenter K. L. (2019). Metabolism and inflammation: implications for traumatic brain injury therapeutics. Expert Rev. Neurother. 19, 227–242. 10.1080/14737175.2019.1582332 30848963

[B15] Lee Y. Morrison B. M. Li Y. Lengacher S. Farah M. H. Hoffman P. N.. (2012). Oligodendroglia metabolically support axons and contribute to neurodegeneration. Nature 487, 443–448. 10.1038/nature11314 22801498 PMC3408792

[B16] Li Z. Du B. Li J. Zhang J. Zheng X. Jia H.. (2017). Cerebrospinal fluid metabolomic profiling in tuberculous and viral meningitis: screening potential markers for differential diagnosis. Clin. Chim. Acta 466, 38–45. 10.1016/j.cca.2017.01.002 28063937

[B17] Ligneul C. Palombo M. Hernández-Garzón E. Carrillo-de Sauvage M. A. Flament J. Hantraye P.. (2019). Diffusion-weighted magnetic resonance spectroscopy enables cell-specific monitoring of astrocyte reactivity *in vivo*. NeuroImage 191, 457–469. 10.1016/j.neuroimage.2019.02.046 30818026

[B18] Lu W. T. Sun S. Q. Li Y. Xu S. Y. Gan S. W. Xu J.. (2019). Curcumin ameliorates memory deficits by enhancing lactate content and MCT2 expression in APP/PS1 transgenic mouse model of Alzheimer's disease. Anat. Rec. 302, 332–338. 10.1002/ar.23969 30312017

[B19] Marais S. Thwaites G. Schoeman J. F. Török M. E. Misra U. K. Prasad K.. (2010). Tuberculous meningitis: a uniform case definition for use in clinical research. Lancet Infect. Dis. 10, 803–812. 10.1016/S1473-3099(10)70138-9 20822958

[B20] Margineanu M. B. Fiumelli H. Mahmood H. Magistretti P. J. (2018). L-lactate regulates the expression of plasticity and neuroprotection genes in cortical neurons: a transcriptome analysis. Front. Mol. Neurosci. 11:375. 10.3389/fnmol.2018.00375 30364173 PMC6191511

[B21] Mason S. (2017). Lactate shuttles in neuroenergetics-homeostasis, allostasis and beyond. Front. Neurosci. 11:43. 10.3389/fnins.2017.00043 28210209 PMC5288365

[B22] Mason S. Reinecke C. J. Kulik W. Van Cruchten A. Solomons R. van Furth A. M. T. (2016). Cerebrospinal fluid in tuberculous meningitis exhibits only the L-enantiomer of lactic acid. BMC Infect. Dis. 16:251. 10.1186/s12879-016-1597-9 27267176 PMC4897924

[B23] Mason S. van Furth A. M. Mienie L. J. Engelke U. F. Wevers R. A. Solomons R.. (2015). A hypothetical astrocyte-microglia lactate shuttle derived from a 1H NMR metabolomics analysis of cerebrospinal fluid from a cohort of South African children with tuberculous meningitis. Metabolomics 11, 822–837. 10.1007/s11306-014-0741-z 26109926 PMC4475545

[B24] Mazuel L. Blanc J. Repond C. Bouchaud V. Raffard G. Déglon N.. (2017). A neuronal MCT2 knockdown in the rat somatosensory cortex reduces both the NMR lactate signal and the BOLD response during whisker stimulation. PLoS ONE 12:e0174990. 10.1371/journal.pone.0174990 28388627 PMC5384673

[B25] Oses J. P. Müller A. P. Strogulski N. R. Moreira J. D. Böhmer A. E. Hansel G. (2019). Sustained elevation of cerebrospinal fluid glucose and lactate after a single seizure does not parallel with mitochondria energy production. Epilepsy Res. 152, 35–41. 10.1016/j.eplepsyres.2019.03.007 30875635

[B26] Pellerin L. Magistretti P. J. (1994). Glutamate uptake into astrocytes stimulates aerobic glycolysis: a mechanism coupling neuronal activity to glucose utilization. PNAS 91, 10625–10629. 10.1073/pnas.91.22.10625 7938003 PMC45074

[B27] Peyron C. Faraco J. Rogers W. Ripley B. Overeem S. Charnay Y.. (2000). A mutation in a case of early onset narcolepsy and a generalized absence of hypocretin peptides in human narcoleptic brains. Nat. Med. 6:991. 10.1038/79690 10973318

[B28] Quintard H. Patet C. Zerlauth J. B. Suys T. Bouzat P. Pellerin L.. (2016). Improvement of neuroenergetics by hypertonic lactate therapy in patients with traumatic brain injury is dependent on baseline cerebral lactate/pyruvate ratio. J. Neurotrauma 33, 681–687. 10.1089/neu.2015.4057 26421521 PMC4827289

[B29] Rinholm J. E. Hamilton N. B. Kessaris N. Richardson W. D. Bergersen L. H. Attwell D. (2011). Regulation of oligodendrocyte development and myelination by glucose and lactate. J. Neurosci. 31, 538–548. 10.1523/JNEUROSCI.3516-10.2011 21228163 PMC3044866

[B30] Rosko L. Smith V. N. Yamazaki R. Huang J. K. (2018). Oligodendrocyte bioenergetics in health and disease. Neuroscientist. 25, 334–343. 10.1177/1073858418793077 30122106 PMC6745601

[B31] San Martín A. Arce-Molina R. Galaz A. Pérez-Guerra G. Barros L. F. (2017). Nanomolar nitric oxide concentrations quickly and reversibly modulate astrocytic energy metabolism. J. Biol. Chem. 292, 9432–9438. 10.1074/jbc.M117.777243 28341740 PMC5454122

[B32] Schousboe A. Sonnewald U. Waagepetersen H. S. (2003). Differential roles of alanine in GABAergic and glutamatergic neurons. Neurochem. Int. 43, 311–315. 10.1016/S0197-018600017-2 12742074

[B33] Siddiqi Z. Fatma J. Karoli R. Siddiqi M. S. Singhal V. Gupta M. (2018). Cerebrospinal fluid lactate in tubercular meningitis: diagnostic or prognostic marker. J. Assoc. Physicians India 66, 722–725. 10.4103/0028-3886.232330 30477060

[B34] Tauffenberger A. Fiumelli H. Almustafa S. Magistretti P. J. (2019). Lactate and pyruvate promote oxidative stress resistance through hormetic ROS signaling. Cell Death Dis. 10, 1–16. 10.1038/s41419-019-1877-6 31506428 PMC6737085

[B35] Thannickal T. C. Moore R. Y. Nienhuis R. Ramanathan L. Gulyani S. Aldrich M.. (2000). Reduced number of hypocretin neurons in human narcolepsy. Neuron 27, 469–474. 10.1016/S0896-627300058-1 11055430 PMC8760623

[B36] Valerio A. Nisoli E. (2015). Nitric oxide, interorganelle communication, and energy flow: a novel route to slow aging. Front. Cell Dev. Biol. 3:6. 10.3389/fcell.2015.00006 25705617 PMC4319459

[B37] Vohra R. Aldana B. I. Bulli G. Skytt D. M. Waagepetersen H. Bergersen L. H.. (2019). Lactate-mediated protection of retinal ganglion cells. J. Mol. Biol. 431, 1878–1888. 10.1016/j.jmb.2019.03.005 30878479

[B38] Vohra R. Kolko M. (2020). Lactate: more than merely a metabolic waste product in the inner retina. Mol. Neurobiol. 57, 2021–2037. 10.1007/s12035-019-01863-8 31916030

[B39] Wolahan S. M. Mao H. C. Real C. Vespa P. M. Glenn T. C. (2018). Lactate supplementation in severe traumatic brain injured adults by primed constant infusion of sodium L-lactate. J. Neurosci. Res. 96, 688–695. 10.1002/jnr.24085 28543565 PMC5696121

[B40] Zhang P. Zhang W. Lang Y. Qu Y. Chen J. Cui L. (2019). 1H nuclear magnetic resonance-based metabolic profiling of cerebrospinal fluid to identify metabolic features and markers for tuberculosis meningitis. Infect. Genet. Evol. 68, 253–264. 10.1016/j.meegid.2019.01.003 30615950

[B41] Zheng Y. Wang X. M. (2018). Expression changes in lactate and glucose metabolism and associated transporters in basal ganglia following hypoxic-ischemic reperfusion injury in piglets. Am. J. Neuroradiol. 39, 569–576. 10.3174/ajnr.A5505 29326137 PMC7655301

[B42] Zhou Q. Zheng H. Chen J. Li C. Du Y. Xia H.. (2018). Metabolic fate of glucose in the brain of APP/PS1 transgenic mice at 10 months of age: a 13C NMR metabolomic study. Metab. Brain Dis. 33, 1661–1668. 10.1007/s11011-018-0274-7 29946959

[B43] Zimmer E. R. Parent M. J. Souza D. G. Leuzy A. Lecrux C. Kim H. I.. (2017). [18F] FDG PET signal is driven by astroglial glutamate transport. Nature Neurosci. 20, 393–395. 10.1038/nn.4492 28135241 PMC5378483

[B44] Zulfiqar S. Garg P. Nieweg K. (2019). Contribution of astrocytes to metabolic dysfunction in the Alzheimer's brain. Biol. Chem. 400, 1113–1127. 10.1515/hsz-2019-0140 31188740

